# Magnetic resonance imaging reproducibility for rotator cuff partial tears in patients up to 60 years

**DOI:** 10.1186/s12891-019-2760-4

**Published:** 2019-08-21

**Authors:** João Alberto Yazigi Junior, Fábio Anauate Nicolao, Nicola Archetti Netto, Fabio Teruo Matsunaga, Jéssica Hae Lim Lee, Stéphanie Yuri Torres Ogata, Leonardo Massamaro Sugawara, André Yui Aihara, Marcel Jun Sugawara Tamaoki

**Affiliations:** 1 Orthopedics and Traumatology - Division of Hand Surgery and Upper Limb, Federal University of São Paulo (UNIFESP/EPM), Borges Lagoa Road, 776, São Paulo, 04038-030 Brazil; 2 Department of Radiology, Federal University of São Paulo (UNIFESP/EPM), Borges Lagoa Road, 776, São Paulo, 04038-030 Brazil

**Keywords:** Rotator cuff, Magnetic resonance imaging, Reproducibility of results

## Abstract

**Background:**

Magnetic resonance imaging (MRI) is the gold standard in diagnosing rotator cuff pathology; however, there is a lack of studies investigating the reliability agreement for supraspinatus partial-thickness tears among orthopaedic surgeons and musculoskeletal (MSK) radiologists.

**Methods:**

Sixty digital MRI scans (1.5 Tesla) were reviewed by two orthopaedic shoulder surgeons, two MSK radiologists, two fellowship-trained shoulder surgeons, and two fellowship-trained orthopaedic surgeons at two distinct times. Thirty-two scans of partial-thickness tears and twenty-eight scans of the supraspinatus tendon with no tears were included. Supraspinatus tendonosis and tears, long head of the biceps pathology, acromial morphology, acromioclavicular joint pathology and muscle fatty infiltration were assessed and interpreted according to the Goutallier system. After a four-week interval, the evaluators were asked to review the same scans in a different random order. The statistical analyses for the intra- and interobserver agreement results were calculated using the kappa value and 95% confidence intervals.

**Results:**

The intraobserver agreement for supraspinatus tears was moderate among the MSK radiologists (k = 0.589; 95% CI, 0.446–0.732) and the orthopaedic shoulder surgeons (k = 0.509; 95% CI, 0.324–0.694) and was fair among the fellowship-trained shoulder surgeons (k = 0.27; 95% CI, 0.048–0.492) and the fellowship-trained orthopaedic surgeons (k = 0.372; 95% CI, 0.152–0.592). The overall intraobserver agreement was good (k = 0.627; 95% CI, 0.576–0.678). The intraobserver agreement was moderate for biceps tendonosis (k = 0.491), acromial morphology (k = 0.526), acromioclavicular joint arthrosis (k = 0.491) and muscle fatty infiltration (k = 0.505). The interobserver agreement results for supraspinatus tears were fair and poor among the evaluators: the MSK radiologists and the orthopaedic shoulder surgeons had the highest agreement (k = 0.245; 95% CI, 0.055–0.435).

**Conclusions:**

In this sample of digital MRI scans, there was an overall good intraobserver agreement for supraspinatus partial tears; however, there were also poor and fair interobserver agreement results. The evaluators with higher levels of experience (the orthopaedic shoulder surgeons and the MSK radiologists) demonstrated better results than evaluators with lower levels of experience.

**Electronic supplementary material:**

The online version of this article (10.1186/s12891-019-2760-4) contains supplementary material, which is available to authorized users.

## Background

Magnetic resonance imaging (MRI) is the gold standard for evaluating rotator cuff tears (RCTs), providing information that is often not diagnosed on clinical examination and other complementary shoulder exams such as ultrasonography; however, the reliability of the diagnosis and the classification of some lesions varies according to the level of experience of the evaluator [1–6].

Although prior studies have observed high agreement among radiologists and orthopaedists in diagnosing rotator cuff full-thickness tears and tendon retraction according to MRI scans, there is a lack of studies that have investigated intra- and interobserver agreement among orthopaedic surgeons and musculoskeletal (MSK) radiologists for partial-thickness tears [7–11]. One study assessed interobserver agreement among ten fellowship-trained orthopaedic shoulder surgeons for supraspinatus partial-thickness tears, indicating poor agreement in predicting the grade of this type of lesion (k = − 0.11) [3]. In addition, the first published studies evaluating agreement for the diagnosis of RCTs were performed only among radiologists who evaluated images from older MRI scanning devices, a factor that may have influenced the final outcome [7, 10, 11]. In one study, images were evaluated by four independent observers who used only their discretion and experience and had not received adequate training for analysing the scans [10].

MRI evaluation is common practice for orthopaedic surgeons and MSK radiologists; therefore, evaluating the reproducibility of MRI for diagnosing supraspinatus partial-thickness tears is important for determining the reliability of this diagnostic test [12, 13].

The purpose of this study was to determine intra- and interobserver agreement among orthopaedic surgeons and MSK radiologists in diagnosing supraspinatus partial-thickness tears and associated pathologies. Our hypothesis was that evaluators with a higher level of experience would present better agreement results than evaluators with a lower level of experience.

## Methods

A single-centre study was performed using digital MRI scans (1.5 Tesla and a dedicated transmit-receive shoulder coil) of sixty patients with shoulder pain from April to May 2017 (in the Diagnostics of America SA, Brazil): these included T1- and T2-weighted scans with axial, oblique coronal and sagittal sections. Institutional ethics approval was obtained before the study initiation (No. 0108/2017) by the Federal University of São Paulo. The inclusion criteria for the study were MRI scans of patients of both sexes aged 18 to 60 years with complaints of shoulder pain. Patients with previous shoulder surgery and severe osteoarthritis and with images that consisted of artefacts or images that might prevent the proper evaluation of rotator cuff tendons (low definition, tremors), as well as the absence of any T1- or T2-weighted MRI scans, were excluded. The 60 included MRI scans were previously selected by an independent MSK radiologist who did not participate as an observer: 20 scans from patients aged between 30 and 40 years, 20 from patients aged between 40 and 50 years, and 20 from patients aged between 50 and 60 years. Thirty-two scans of partial-thickness tears and twenty-eight scans of supraspinatus tendons with no tears were included.

The patient demographics were as follows: 30 males (50%) and 30 females (50%) with a mean age of 44 years (range: 30 to 58 years). Scans were randomly numbered (from 1 to 60) and were free of any identifying information to ensure patient confidentiality.

The patients were scanned in a supine position with slight elevation of the contralateral side with the use of a 1.5 Tesla MRI device, and the ipsilateral side was positioned with the body in slight external rotation. The shoulder studied was positioned as centrally as possible.

Three sections were assessed in the T2-weighted scans: an axial, an oblique coronal and an oblique sagittal plane perpendicular to the supraspinatus fossa. In each plane, 16 to 20 cuts were acquired in the T2-weighted scans, and each cut had a 4-mm section thickness and a 0.4-mm gap. In the T1-weighted scans, 2 planes with fat suppression were obtained with a 4-mm thickness and a 0.3-mm gap centred on the rotator cuff muscles: a coronal oblique plane and a sagittal plane with 12 to 16 cuts each.

The scans were analysed by two orthopaedic shoulder surgeons (with 10 and 15 years of experience), two MSK radiologists (with 6 and 10 years of experience), two orthopaedic fellowship-trained shoulder surgeons, and two fellowship-trained orthopaedics; the two-step analyses occurred 4 weeks apart at a single location to help reduce recall bias. All recruited orthopaedic shoulder surgeons and MSK radiologists for analysis had practiced for a minimum of 5 years and had completed at least a 1-year fellowship. A training phase was performed for the standard evaluation of tendonosis, supraspinatus tears (identified by fluid signal intensity in T2-weighted coronal and sagittal scans), acromial morphology, long head of the biceps pathology, acromioclavicular joint pathology and muscle fatty infiltration.

All participants agreed and signed the informed consent form, and everyone involved was informed about the prognoses, possible complications and study objectives.

Each patients’ MRI images were randomized sequentially using the computer program randomizer (www.randomizer.org) after the first analysis by an orthopaedic surgeon who did not evaluate those images. All images were inspected with coronal, sagittal and axial cuts in the T1-weighted sequences and with sagittal cuts in the T2-weighted sequences. Any identification of the patients was concealed from the observers.

**Fig. 1:**
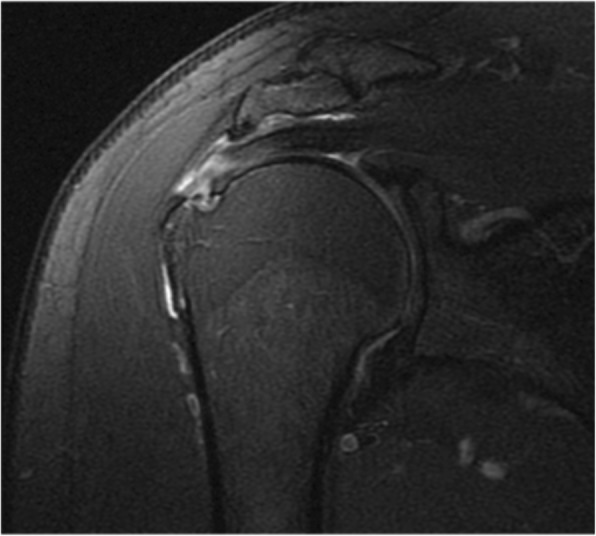
Coronal view of supraspinatus partial-thickness tear

**Fig. 2:**
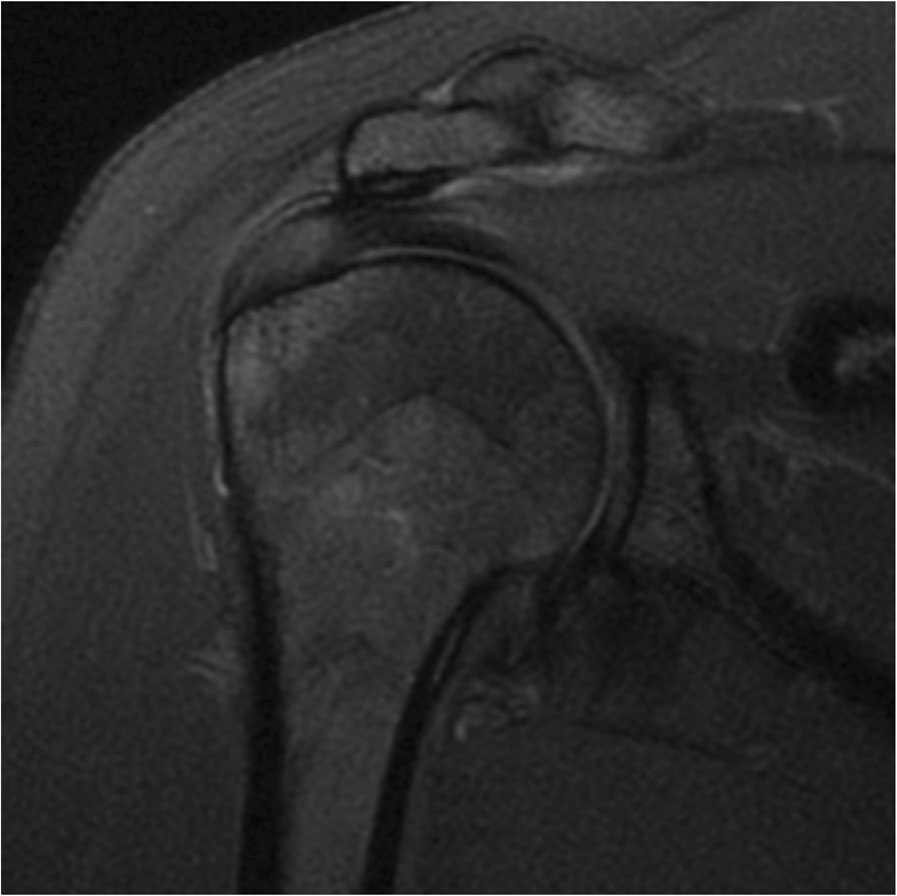
Focal tendonosis on the supraspinatus tendon

**Fig. 3:**
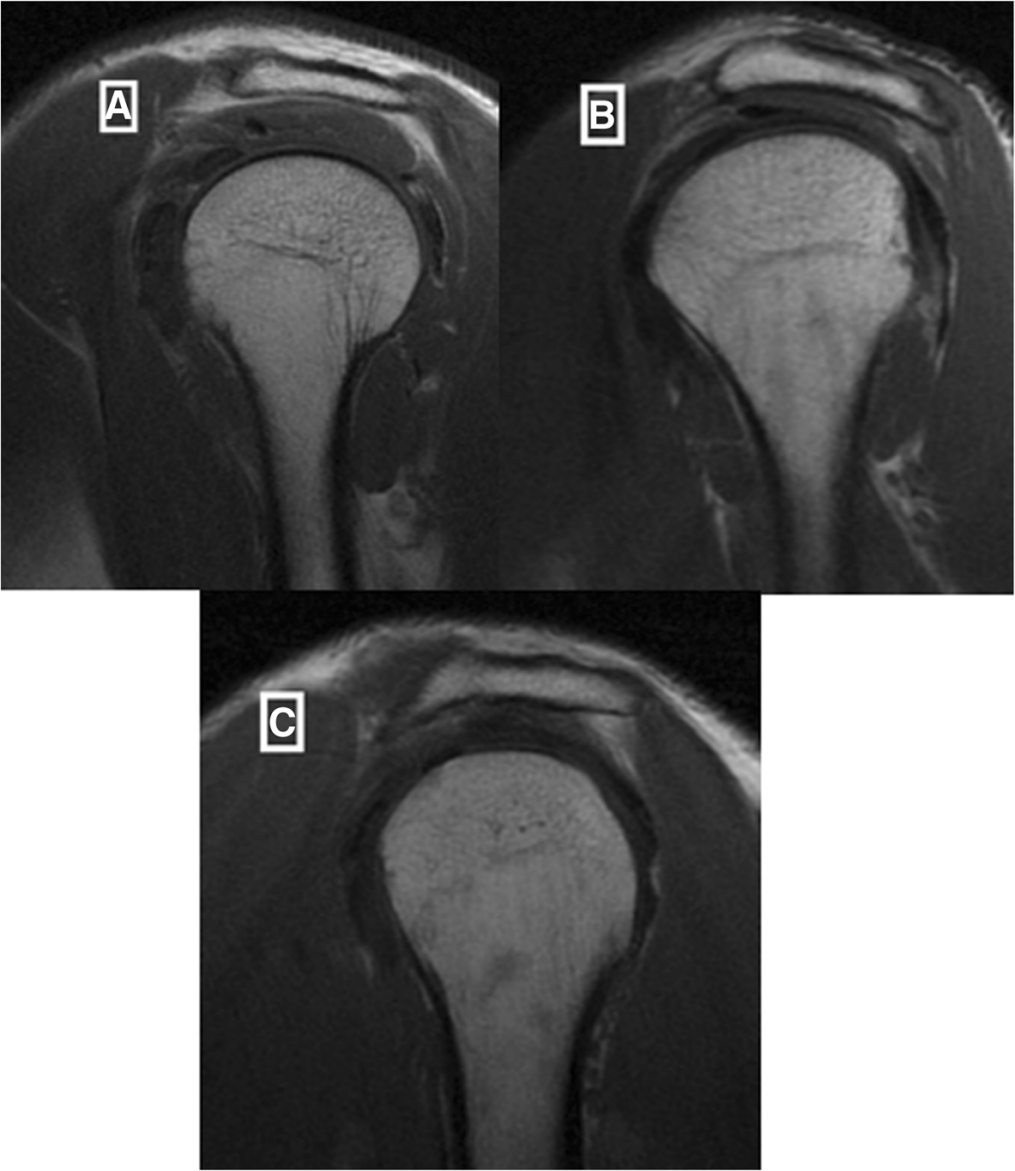
Acromial morphology in the sagittal plane in a T1-weighted sequence: flat (**a**), curved (**b**) and hooked (**c**)

The evaluators completed the evaluation form developed by the authors for this study (see Additional file 1): supraspinatus tendonosis and tears (Figures 1 and 2); long head of the biceps pathology (tendonosis, subluxation or medial luxation and tears); acromial morphology in the sagittal plane in T1-weighted images (Figure 3), which was the plane that demonstrated the largest curvature of the acromion (flat, curved or hooked according to Bigliani’s classification); acromioclavicular (AC) joint pathology (arthrosis, spurs and osseous oedema); and muscle fatty infiltration according to the Goutallier system classification for rotator cuff degeneration on sagittal, T1-weighted images [14, 15].

### Statistical analyses

To evaluate the intra- and interobserver agreement variability, the kappa value was used for each variable studied among orthopaedics and MSK radiologists, with a 95% confidence interval.

Data were analysed in Microsoft Office Excel 2007 using 2 × 2 contingency tables. The values were interpreted according to the adapted guidelines of Landis and Koch. Excellent agreement occurred when the kappa value was between 0.81 and 1.00; good agreement between 0.61 and 0.80; moderate agreement between 0.41 and 0.60; fair agreement between 0.21 and 0.40; and poor agreement less than 0.20 [16, 17].

## Results

Four digital MRI scans were excluded: three had no T1-weighted sagittal cuts, and one was taken after the RCT repair; therefore, fifty-six scans were used for the evaluations.

There were several statistically significant instances (*p* < 0.05) of intraobserver agreement. For supraspinatus tears, the intraobserver agreement among the shoulder surgeons (k = 0.509; 95% CI, 0.324–0.694) and the MSK radiologists (k = 0.589; 95% CI, 0.446–0.732) was moderate, and the intraobserver agreement was fair among the fellowship-trained orthopaedic surgeons (k = 0.372; 95% CI, 0.152–0.592) and the orthopaedic fellowship-trained shoulder surgeons (k = 0.27; 95% CI, 0.048–0.492). The overall intraobserver agreement for supraspinatus partial-thickness tears was good (k = 0.627; 95% CI, 0.576–0.678). For supraspinatus tendonosis, the overall intraobserver agreement was moderate (k = 0.437; 95% CI, 0.345–0.529): the intraobserver agreement was fair among the fellowship-trained shoulder surgeons (k = 0.297; 95% CI, − 0.058-0.652) and moderate among the orthopaedic fellowship-trained surgeons (k = 0.419; 95% CI, 0.209–0.629) and the shoulder surgeons (k = 0.494; 95% CI, 0.273–0.715), with the highest result, classified as good, observed among the MSK radiologists (k = 0.601; 95% CI, 0.335–0.867) (Table 1).

**Table 1:** Intraobserver reliability of the supraspinatus lesions

	Supraspinatus			
	Tendonosis		Tears	
	k	p	k	p
Shoulder Surgeons	0.494	< 0.001	0.509	< 0.001
Shoulder Fellowships	0.297	0.001	0.270	0.002
MSK Radiologists	0.601	< 0.001	0.589	< 0.001
Orthopaedic Fellowships	0.419	< 0.001	0.372	< 0.001
Overall	0.437	< 0.001	0.627	< 0.001

The intraobserver agreement was good among the MSK radiologists for long head of the biceps tendonosis (k = 0.64; 95% CI, 0.497–0.783), and the intraobserver agreement was good among the shoulder surgeons for long head of the biceps subluxation (k = 0.663; 95% CI, 0.043–1) and excellent for long head of the biceps tears (k = 1.0; 95% CI, 1.0). There was good intraobserver agreement among the MSK radiologists when evaluating AC joint arthrosis (k = 0.737; 95% CI, 0.613–0.861) and among the shoulder surgeons when evaluating osseous oedema (k = 0.674; 95% CI, 0.496–0.852). The overall intraobserver agreement was fair for AC joint spurs (k = 0.294; 95% CI, 0.225–0.363) and moderate for acromial morphology (k = 0.526; 95% CI, 0.476–0.576) and muscle fatty infiltration (k = 0.505; 95% CI, 0.46–0.55) (Table 2).

**Table 2:** Intraobserver reliability of associated pathologies

	Long head of the biceps pathology						Acromial morphology		AC joint pathology						Muscle fatty infiltration	
	Tendonosis		Subluxation or Medial luxation		Tears				Joint arthrosis		Osseous edema		Spurs		Goutalier	
	k	p	k	p	k	p	k	p	k	p	k	p	k	p	k	p
Shoulder Surgeons	0.384	< 0.001	0.663	< 0.001	1	< 0.001	0.488	< 0.001	0.471	< 0.001	0.674	< 0.001	0.124	0.183	0.385	< 0.001
Shoulder Fellowships	0.299	0.001	0.36	< 0.001	0.491	< 0.001	0.414	< 0.001	0.39	< 0.001	0.444	< 0.001	0.258	0.003	0.242	< 0.001
MSK Radiologists	0.64	< 0.001	0.117	0.149	0.461	< 0.001	0.527	< 0.001	0.737	< 0.001	0.549	< 0.001	0.449	< 0.001	0.351	< 0.001
Orthopaedic Fellowships	0.304	0.001	0.294	0.001	0.241	0.001	0.501	< 0.001	0.339	< 0.001	0.612	< 0.001	0.14	0.12	0.215	< 0.001
Overall	0.491	< 0.001	0.228	< 0.001	0.355	< 0.001	0.526	< 0.001	0.491	< 0.001	0.607	< 0.001	0.294	< 0.001	0.505	< 0.001

The results of interobserver agreement are presented in Table 3. For supraspinatus tears, the results of intraobserver agreement were fair and poor in most evaluations, with the highest result observed among the MSK radiologists and the orthopaedic shoulder surgeons (k = 0.245; 95% CI, 0.055–0.435). The results of intraobserver agreement for acromial morphology and muscle fatty infiltration were poor and fair, respectively, among the evaluators; however, the interobserver agreement results for long head of the biceps tears among the shoulder surgeons and the orthopaedic fellowship-trained shoulder surgeons were good (k = 0.663; 95% IC, 0.043–1).

**Table 3:** Interobserver agreement of supraspinatus tears and associated pathologies

	Supraspinatus				Long head of biceps pathology						Acromial Morphology		AC joint pathology						Muscle fatty infiltration	
	Tendonosis		Tears		Tendonosis		Medial Sub or Luxation		Tears				Arthosis		Spurs		Osseous Edema			
	k	p	k	p	k	p	k	p	k	p	k	p	k	p	k	p	k	p	k	p
Shoulder surgeons/Shoulder Fellowships	0.152	0.038	0.094	0.057	0.128	0.044	0.168	0.001	0.663	< 0.001	−0.015	0.393	0.237	0.012	0.162	0.011	0.104	0.184	0.044	0.13
Shoulder surgeons/MSK Radiologists	0.204	0.028	0.245	0.009	0.209	0.006	0.21	< 0.001	0.061	0.059	0.046	0.011	0.306	0.001	0.125	0.09	0.171	0.005	0.217	0.021
Shoulder surgeons/Orthopaedic Fellowships	0.325	0.001	0.094	0.057	0.062	0.331	0.09	0.021	0.05	0.087	0.034	0.059	0.389	< 0.001	−0.034	0.681	0.099	0.182	0.038	0.151
MSK Radiologists/Shoulder Fellowships	0.226	0.006	0.109	0.052	0.281	0.002	0.156	0.095	0.043	0.338	−0.149	0.039	0.202	0.028	0.106	0.249	0.318	< 0.001	0.01	0.709
MSK Radiologists/Orthopaedic Fellowships	0.027	0.77	0.080	0.154	0.211	0.021	0.147	0.084	0.271	0.004	0.125	0.094	0.207	0.025	0.105	0.254	0.354	< 0.001	0.012	0.616
Shoulder Fellowships/Orthopaedic Fellowships	0.04	0.577	0.238	0.012	0.347	< 0.001	0.355	< 0.001	0.032	0.427	0.056	0.432	0.132	0.123	0.145	0.094	0.34	< 0.001	0.139	< 0.001

## Discussion

This study was performed to assess inter- and intraobserver agreement among experienced orthopaedic shoulder surgeons, MSK radiologists, orthopaedic fellowship-trained shoulder surgeons, and fellowship-trained orthopaedic surgeons for supraspinatus partial-thickness tears and associated pathologies: long head of the biceps pathology, acromial morphology, AC joint pathology, and muscle fatty infiltration. The overall intraobserver agreement was good (k = 0.627) for supraspinatus tears and moderate for tendonosis (k = 0.437). As we hypothesized, the evaluators with higher levels of experience (the orthopaedic shoulder surgeons and the MSK radiologists) demonstrated higher inter- and intraobserver agreement results than the evaluators with lower levels of experience. The best interobserver agreement results for supraspinatus tears were found among the orthopaedic shoulder surgeons and MSK radiologists (k = 0.245).

The strengths of this study were that a sample of sixty digital shoulder MRI scans were used, which is larger than the sample size of many published studies on this topic; in addition, intra- and interobserver agreement was evaluated among orthopaedic shoulder surgeons, MSK radiologists, orthopaedic fellowship-trained shoulder surgeons, and fellowship-trained orthopaedic surgeons, allowing a comparison among four groups of evaluators with different levels of experience [3, 8, 9, 18]. The weaknesses of the study were that no other RCTs (infraspinatus and subscapularis tears) were evaluated, the grade of tendonosis and the grade of supraspinatus partial-thickness tear were not evaluated, and there were no comparisons performed between MRI arthrography or arthroscopy, which would provide more reliability to our study [1, 19].

Bauer et al. performed a reliability study with 3.0 Tesla digital MRI scans among three experienced MSK radiologists for supraspinatus tendonosis and partial-thickness tears, grading the tendonosis and the tear size. In their study, there were good to excellent interobserver kappa values. The inter- and intraobserver results for tendonosis and partial-thickness tears were higher in the study performed by Bauer et al. than in our study, probably because we also included scans of patients without rotator cuff lesions, and our scans were performed on a 1.5 Tesla digital MRI device [1].

Similar to other studies, we conducted an instructional scoring questionnaire and provided the evaluators with prior training, allowing a standardized assessment of scans with coronal, sagittal and axial cuts in T1- and T2-weighted sequences [4, 9]. Although we conducted a training and standardization for evaluation of the exams, the interobserver agreement results were fair and poor in most evaluations. We expected, based on the results of the literature, that these results would be better than the results we found [1, 9]. The evaluators with high levels of experience demonstrated the best agreement results, showing that in addition to the training, the evaluator experience was relevant for the results.

Other studies evaluated the interobserver agreement for pathologies associated with RCTs among orthopaedic shoulder surgeons and found poor agreement results for acromion morphology (k = 0.06), which is similar to that we found in the present study [4, 9]. In this study, there were good and excellent intraobserver agreement results for long head of the biceps and AC pathologies among the shoulder surgeons and the MSK radiologists; however, there were moderate agreement results for muscle fatty infiltration.

One study evaluated the interobserver reliability among three shoulder surgeons for patients who underwent arthroscopic rotator cuff repair and had preoperative MRI scans and found a moderate interobserver agreement for rotator cuff degeneration using the Goutallier classification [20]. In our study, the interobserver agreement results were fair and poor; this difference probably occurred because the Lippe et al. study had more patients than our study with advanced muscle fatty infiltration. In our study, most patients had no fatty infiltration or mild grades of fatty infiltration.

In the present study, the agreement results for supraspinatus tears and associated pathologies were evaluated among observers with different levels of experience, reproducing situations of daily clinical practice among orthopaedic surgeons and MSK radiologists. Some of the results of the present study were consistent with the results of previous studies, but others were not. The authors intend to conduct future studies of reliability for other shoulder pathologies, such as SLAP and Bankart lesions, and to perform future evaluations of comparisons of MRI arthrography and surgery.

## Conclusion

In this sample of digital MRI scans, there was an overall good intraobserver agreement for supraspinatus partial tears; however, there were also poor and fair interobserver agreement results. The evaluators with higher levels of experience (the orthopaedic shoulder surgeons and the MSK radiologists) demonstrated better intra- and interobserver agreement results than the evaluators with lower levels of experience.

## Additional file

Additional file 1: Evaluation form used to perform the analyses of the MRI scans (DOC 1997 kb)

## Data Availability

Data in contingency tables and the dataset are available from the corresponding author at junioryazigi73@yahoo.com.br.

## Abbreviations

MRI: Magnetic resonance imaging
MSK: Musculoskeletal
RCTs: Rotator cuff tears
SLAP: Superior labral antero-posterior
