# Supplementary material for: Magnetic resonance imaging reproducibility for rotator cuff partial tears in patients up to 60 years
Source: BMC Musculoskelet Disord. 2019 Aug 21;20:383. doi: 10.1186/s12891-019-2760-4 (PMC6702712; doi:10.1186/s12891-019-2760-4)
Supplement: Supplementary file 1 — Evaluation form used to perform the analyses of the MRI scans (DOC 1997 kb) [file 12891_2019_2760_MOESM1_ESM.doc]

**Appendix 1.** Evaluation form used to perform the analyses of the MRI scans

Evaluator name:_________________ Date:__________________

Supraspinatus tendon:

1. There is tendonosis: ( ) Yes / ( ) No
2. There is tears: ( ) Yes / ( ) No

Long head of the biceps pathology: to determine if there is pathology in the long head, use the T2 axial and sagital views:

3) There is tendonosis: ( ) Yes / ( ) No

4) There is subluxation or medial luxation: ( ) Yes / ( ) No

5) There is tears: ( ) Yes / ( ) No

6) Acromial morphology (on the T1 sagittal view pick the cut that demonstrates the maximum amount of acromial curvature):

( ) Flat / ( ) Curved / ( ) Hooked

AC joint pathology :

7) There is AC joint arthrosis: defined by increased signal on the T2 coronal and sagittal views

( ) Yes / ( ) No

8) There is osseous edema: superior or inferior bone proliferation, subchondral cysts and capsuloligamentous thickening seen on the T2 coronal, axial and sagittal views

( ) Yes / ( ) No

9) There is spurs: seen on the T1 coronal and sagittal views

( ) Yes / ( ) No

10) Classify the quality of the muscle fatty infiltration according to the Goutallier system.

( ) Grade 0 (none)

( ) Grade 1 (Streaks of fatty tissue)

( ) Grade 2 (More muscle than fat)

( ) Grade 3 (Equal amounts of muscle and fat)

( ) Grade 4 (More fat than muscle)
